# A single-center study: three years of experience with whole-exome sequencing in diagnosing pediatric hematological disorders

**DOI:** 10.1186/s13052-026-02268-9

**Published:** 2026-05-09

**Authors:** Shaimaa Salah, Amira Mobarak, Saleh Nouh Alshanbari, Asem A. Bukhari, Fouzia Alsobhi, Sally Abdelazim Ali Farahat, Hassan Musa Masmali

**Affiliations:** 1 Pediatric Department, Faculty of Medicine, Kafrelsheikh University, Kafr El-Shaikh, Egypt; 2 Pediatrics Department, Faculty of Medicine, Tanta University, Tanta, Egypt; 3 Maternity and Children Hospital, Makkah, Saudi Arabia

**Keywords:** WES, Pediatric, Blood, Genetic testing

## Abstract

**Background:**

Blood disorders in children can present with a wide range of nonspecific symptoms, which may overlap with other conditions. Traditional diagnostic methods can sometimes struggle to identify the underlying cause. Whole-exome sequencing (WES)—is increasingly recognized as a valuable diagnostic tool, as early genetic diagnosis can offer clarity, enabling more personalized treatment approaches and improving prognostic accuracy.

**Methods:**

Between January 2022 and December 2024, 67 patients at the pediatric hematology department of Maternity and Children Hospital in Makkah, Saudi Arabia, underwent WES after conventional diagnostic methods failed to provide definitive diagnoses. We retrospectively analysed their results.

**Results:**

The study included 67 patients with an average age of 6.5 ± 4.6 years (range 0.1–18 years), 53% of whom were male. The majority (88%) were of Saudi descent. WES provided a molecular diagnosis for 49% (*n* = 33) of the patients, identifying pathogenic or likely pathogenic variants. In 11% (*n* = 8) of the patients, no variants were found, whereas 38% (*n* = 26) had variants of uncertain significance in phenotype-related genes. A nonhematological diagnosis was given to 28% of the patients.

**Conclusion:**

Whole-exome sequencing is a valuable tool for diagnosing challenging pediatric blood disorders. The findings also underscore its importance in identifying complex or multifactorial disorders.

## Background

Diagnosing certain hematological disorders can be challenging because overlapping clinical features complicate initial assessments. Whole-exome sequencing (WES) has gained significant traction in clinical practice over the past decade, serving as an indispensable diagnostic tool because of its high throughput and rich informational content. The accumulation of molecular data from diverse populations expands the potential applications of this information. The reported diagnostic yield for WES varies across different studies, ranging from 25% to 52% [1]. 

Identifying the specific genetic causes associated with an individual’s health condition allows clinicians to customize treatment strategies on the basis of the patient’s genetic makeup and provides essential information for genetic counseling of the family, including recurrence risk assessment and informed reproductive decision-making. Furthermore, WES contributes to the discovery of new candidate genes that can aid in predicting disease and understanding disease progression, thereby advancing personalized healthcare initiatives [2]. 

In this study, we evaluated the diagnostic effectiveness of WES for patients presenting with symptoms and/or signs suspicious of hematological disorders, alongside conventional laboratory tests.

## Methodology

### Study design and participants

This retrospective cohort study evaluated the diagnostic utility of whole exome sequencing (WES) in pediatric patients (aged 1–18 years) presenting with ambiguous hematological findings, in whom conventional diagnostic methods — including clinical evaluation, laboratory testing (e.g., complete blood count, peripheral blood smear, bone marrow examination, coagulation studies, factor assays, hemoglobin electrophoresis, iron studies, basic immunological tests) — had failed to establish a definitive diagnosis. We reviewed the electronic medical records (EMRs) of 67 patients referred to the pediatric hematology department at Maternity and Children Hospital (MCH), Makkah, Saudi Arabia (SA), from 2022 to 2024. Ethical approval was granted by the institutional review board (IRB) of Makkah (H-02–K-076).

Genetic samples (blood or buccal mucosa) were collected according to laboratory recommendations and sent to two specialized commercial laboratories, Raidx and PerkinElmer, for sequencing. Clinical data, including patient demographics, presentations, laboratory results, and WES findings, were extracted from EMRs, after informed consent/ permission was obtained from families/ caregivers, and analysed.

The data were summarized via counts and percentages to represent the frequency of genetic findings.

## Genetic testing

### Revvity methodology

WES was performed on genomic DNA via a capture method, and the DNA was sequenced on the Illumina next-generation sequence (NGS) platform (2 × 150 bp reads). Exons were considered fully covered with ≥ 20X coverage, and variants were reviewed with ≥8X coverage and an alternate allele frequency ≥ 20%. Mitochondrial DNA and copy number variations (CNVs) were also analysed excluding certain genomic regions. The data were analysed via Illumina bcl2fastq, DRAGEN Bio-IT, SnpEff, PerkinElmer ODIN, and BioDiscovery NxClinical software.

### AiLife methodology

WES at AiLife used a paired-end pre-capture library procedure. Sequencing was performed on an NGS platform with ≥ 94% bases covered at ≥ 20X. The variant analysis included single nucleotide variants (SNVs), small indels, and CNVs affecting three or more exons. The data were analysed with BWA, GATK, and in-house pipelines (A-GPS^®^, I-GPS^®^, D-GPS^®^).

Variants were classified per American College of Medical Genetics (ACMG) guidelines as pathogenic (P), likely pathogenic (LP), or variant of unknown significance (VUS). Only these variants were included in the final report. The interpretation of genes related to the patient’s clinical phenotype was based on the patient’s clinical information.

### Analysis of cases without identified variants

All cases, including those without any reportable variants, were processed through the same pipelines.

- In AiLife, analysis was performed using AilisNGS^®^ with alignment to GRCh37 (hs37d5 decoy), variant calling using Sentieon implementation of BWA-MEM and GATK Haplotype Caller, and CNV detection via Atlas-CNV.
- In Revvity, analysis was performed using Illumina DRAGEN Bio-IT v3.4.12 for secondary analysis and internal ODIN v1.01 for tertiary analysis, with annotation by SnpEff. Variants were filtered and classified according to ACMG/ClinGen guidelines, and negative cases were formally documented as “no reportable variants.”

### Pipelines used for secondary analyses

- AiLife: Sentieon (BWA-MEM, GATK Haplotype Caller), Atlas-CNV.
- Revvity: DRAGEN Bio-IT Platform (v3.4.12), SnpEff (v5.0), ODIN v1.01, with CNV/AOH assessed by NxClinical 5.1.

### Validation, sensitivity, and specificity

- Both laboratories are CLIA/CAP-certified and validated their assays according to CAP/CLIA standards.
- Reported validation metrics include:
  - SNP and indel sensitivity and specificity > 99% (AiLife, based on NA12878 and clinical samples).
  - 100% concordance in accuracy and reproducibility in validation sets.
  - CNV detection validated (12/12 known cases correctly identified by AiLife; CNVs assessed by NxClinical in Revvity).
  - Minimum coverage thresholds: ≥20X for adequate base/exon coverage; variants with ≥8X coverage and ≥ 20% VAF reviewed (Revvity).
- Sanger confirmation of SNVs/indels is performed at laboratory director discretion.

## Results

### Demographics

The average age of patients who underwent WES was 6.5 ± 4.6 years, with ages ranging from 0.1 to 18 years. In our study cohort, 53% (*n* = 36/67) were males. Among the total cohort, 88% (*n* = 59) were of Saudi descent, whereas 5% (*n* = 4) were Burmese, 3% (*n* = 2) were Egyptian siblings, and 2 patients had an unknown ethnic background.

Among the 67 patients included in the study, 33 (49%) received a molecular genetic diagnosis through singleton WES, which identified P or LP variants. WES did not identify any variants in 11% (*n* = 8) of the cases, whereas 38% (*n* = 26) had VUSs.

### Clinical presentations

#### Neutropenia

In a group of eleven neutropenia patients, two (18%) received a definitive molecular diagnosis, with P or LP variants identified. WES did not reveal any variants in two patients (18%). Three patients had homozygous VUSs in genes associated with their clinical phenotype. Two patients were diagnosed with Klinefelter syndrome, one of whom (P1) also had a mutation in the LIG1 gene. One female patient (P3) with an X-linked recessive (XLR) variant in the WASP gene presented with persistent neutropenia, thrombocytopenia, and giant platelets in the peripheral blood, along with active cellular bone marrow. Skewed X-inactivation testing was not performed for further evaluation. One male patient (P7) had a VUS in the TAZ gene. Two patients (P1, P5) each had a heterozygous VUS in different genes of interest (LIG1, VPS45). However, further testing, including chromosomal microarray (CMA) or Sanger sequencing, was not conducted to detect additional variants; moreover, familial segregation studies were not performed Table 1.

**Table 1:** Genetic testing results and clinical findings in neutropenia patients

	Gene	Gender	Clinical presentation	Variants	ACMG classification	OMIM phenotype	Zygosity	Mode of inheritance	Lab.
P1	LIG1	M	Insulin-dependent diabetes mellitus, chronic neutropenia	c.1306del	VUS	-immunodeficiency, LIG1 is a gene reportedly associated with immunodeficiency and cancer -Klinefelter syndrome	Het	AR	Revvity
^1^P2	NCKAP1L BTK	F	Moderate to severe neutropenia	c.507–11 C > T c.1548G > C (p.Gln516His)	VUS VUS	-Immunodeficiency 72 with autoinflammation -X-linked agammaglobulinemia	Hom Het	AR XLR	AiLife
P3	WASP	F	Persistent neutropenia	c.1060_1062del (p.Pro354del)	VUS	Neutropenia severe congenital X-linked, variant of WAS	Het	XLR	AiLife
P4	CSF3R	F	Persistent neutropenia and recurrent infections	c.1223T > C (p.Leu408Pro)	VUS	Neutropenia severe congenital − 7	Hom	AR	AiLife
P5	VPS45	M	Congenital neutropenia	c.1129 C > A (p.Pro377Thr)	VUS	Neutropenia, severe, congenital, 5, autosomal recessive	Het	AR	Revvity
P6	KRAS	F	Chronic neutropenia and recurrent infections	c.38G > A (p.Gly13Asp)	P	Cardiofaciocutaneous syndrome 2; Noonan syndrome 3; RAS associated autoimmune leukoproliferative disorder	Het	AD	Revvity
P7	TAZ	M	Neutropenia	c.347G > A (p.Gly116Asp)	VUS	Barth syndrome	Hemizygous XL	XL	Revvity
P8	CD55	F	Neutropenia, recurrent severe infections, and protein-losing enteropathy	c.147_148delinsAT (p.Glu50Ter)	LP	Complement hyperactivation angiopathic thrombosis and protein-losing enteropathy	Hom	AR	AiLife

M; male, F; female, AR; autosomal recessive, AD; autosomal dominant, XL; X−linked, XLR; X−linked recessive, Hom; homozygous, Het; heterozygous

^1^ Female patient presented with moderate to severe neutropenia and normal immunoglobulin levels. Further basic investigations, such as ferritin, triglyceride (TG), and IL-2R levels, were not conducted

### Bi/Pancytopenia

Fourteen patients with bi/pancytopenia were tested. Seven patients (50%) received a definitive molecular diagnosis through WES, revealing P or LP variants. Seven patients (50%) had VUSs in genes consistent with their clinical phenotype, requiring further functional or conventional testing to distinguish pathogenic variants from polymorphisms. Among these patients, ten patients (71%) had autosomal recessive (AR) variants, and two (14%) had X-linked (XL) variants. Fanconi anaemia complementation group (FANC) mutations (FANCA, FANCB, FANCD2) were identified in four patients, two of whom (P9, P10) were siblings with the same gene and variant. Dyskeratosis congenita-related gene mutations were identified in three patients (TERT, RETL1, and TINF2); however, none of them exhibited the full clinical phenotype of the disease at the time of testing, except for cytopenia. P21 had bone marrow fibrosis on a bone marrow biopsy and was found to have an HTRA2 gene mutation, which could explain the neutropenia but not the myelofibrosis (Table 2).

**Table 2:** Genetic testing results and clinical findings in patients with cytopenia

	Gene	Gender	Clinical presentation	Variant	ACMG classification	OMIM phenotype	Zygosity	Mode of inheritance	Lab
P9	FANCA	M	Bicytopenia, Microcephaly, and horseshoe kidney	c.2994T > G (*p*.Tyr998Ter)	LP	Fanconi anemia complementation group A	Hom	AR	AiLife
P10	FANCA	F	Thrombocytopenia and horseshoe kidney	c.2994T > G (p.Tyr998Ter)	LP	Fanconi anemia complementation group A	Hom	AR	AiLife
^1^P11	MYSM1 WAS	M	Neutropenia and thrombocytopenia	c.1561 C > T (p.Gln521Ter) c.1060_1062del (p.Pro354del)	LP VUS	Bone marrow failure syndrome 4 -Neutropenia severe congenital X-linked - Thrombocytopenia X-linked - Wiskott-Aldrich syndrome	Hom Hemizygous	AR XLR	AiLife
P12	FANCD2	F	Neutropenia thrombocytopenia and dysmorphism	c.4175G > C (p.Arg1392Pro)	VUS	Fanconi anemia complementation group D2	Hom	AR	AiLife
P13	TERT	M	Bicytopenia, delayed motor development	c.25G > A (p.Ala9Thr),	VUS	Dyskeratosis congenita	Hom	AR	AiLife
P14	MPIG6B	F	Pancytopenia	c.523 C > T (p.Arg175Ter)	LP	Thrombocytopenia anemia and myelofibrosis	Hom	AR	AiLife
P15	CA2	M	Anemia And thrombocytopenia	c.232 + 1G > A	P	Osteopetrosis	Hom	AR	AiLife
P16	RTEL1	F	Pancytopenia and Bone marrow hypercellularity	c.686G > A (p.Arg229Lys)	VUS	Dyskeratosis congenita autosomal dominant 4	Het	AD	AiLife
^2^P17	FANCB	M	Anemia followed by thrombocytopenia and hypothyroidism	c.952–13 C > T	VUS	Fanconi anemia, complementation group B	Hemizygous	XL	Revvity
^3^P18	TINF2	M	Anemia Thrombocytopenia and bleeding	c.1082T > C (p.Met361Thr)	VUS	Dyskeratosis congenita, autosomal dominant 3	Hom	AD	Revvity
P19	CNV (22q11.21, TBX1) ARSB	M	Pancytopenia, hepatosplenomegaly (HSM), and bleeding in a clinically diagnosed diGeorge syndrome	Seq[GRCh37/hg19] deletion c.1534G > A (p.Val512Met),	P VUS	DiGeorge syndrome Mucopolysaccharidosis (MPS) type VI (Maroteaux-Lamy)	Het Hom	AD AR	AiLife
P20	STXBP2	M	Clinical criteria of hemophagocytic lymphohistiocytosis and a history of infant sibling death	c.698T > G (p.Met233Arg)	VUS	Hemophagocytic lymphohistiocytosis familial 5 with or without microvillus inclusion disease	Hom	AR	AiLife
P21	HTRA2	M	Pancytopenia and myelofibrosis in BM Biopsy	c.1212-17T > C	VUS	3-methylglutaconic aciduria type VIII	Hom	AR	AiLife
P22	KRAS	F	Bicytopenia, hypothyroidism, splenomegaly	c.37G > T (p.Gly13Cys)	P	Cardiofaciocutaneous syndrome 2; Noonan syndrome 3; RAS associated autoimmune leukoproliferative disorder	Mosaic	AD	Revvity

^1^A male patient who presented with thrombocytopenia and neutropenia was found to have two variants in 2 separate genes

^2^A male patient with neutropenia and thrombocytopenia later progressed to acute myeloid leukemia (AML)

^3^ A male patient with anemia and thrombocytopenia responded to steroids and eltrombopag. TINF2 mutations can lead to a broad range of clinical manifestations; therefore, telomere length testing should be used to differentiate pathogenic variants from polymorphic variants, particularly in the absence of functional data

### Bleeding tendency

Seven patients with a bleeding tendency and abnormal coagulation tests underwent WES. WES alone provided a genetic diagnosis in three patients (42%), whereas four (57%) had VUSs in genes consistent with their clinical phenotype (Table 3).

**Table 3:** Genetic testing results and clinical findings in patients with bleeding tendency

	Gene	Gender	Clinical presentation	variant	ACMG classification	OMIM phenotype	zygosity	Mode of inheritance	Lab
P23	KLKB1	F	Bleeding tendency and prolonged PTT	c.870del(*p*.Glu290AspfsTer18)	LP	Fletcher factor (prekallikrein) deficiency	Hom	AR	AiLife
P24	*F12*	F	Bleeding tendency and prolonged PTT	c.396 A > T(p.Lys132Asn)	VUS	Factor XII deficiency	Hom	AR	AiLife
^1^P25	F9 GJB2	M	A known case of hemophilia B, hyperactivity, and delayed speech	c.127 C > T (p.Arg43Trp) c.235del	P VUS	Hemophilia B GJB2 - Related hearing loss	Hemizygous Het	XL AD	Revvity
P26	F9	M	Bleeding tendency and prolonged PTT	c.1113 C > A (p.Tyr371Ter)	P	Hemophilia B	Hemizygous	XL	Revvity
P27	F7	M	Bleeding tendency, mild deficiency of factor 7, and bilateral hearing loss	c.356G > T (p.Gly119Val)	VUS	Factor VII deficiency	Het	AR	AiLife
^2^P28	CD36	F	Bleeding disorder and subdural hematoma	c.379_381dup	VUS	Platelet glycoprotein IV deficiency	Het	AR	Revvity

^1^A male patient with hemophilia B, along with signs of hyperactivity and delayed speech, was found to have a pathogenic variant in the GJB2 gene, which is associated with hearing loss

^2^A female patient who has a severe bleeding tendency and a heterozygous variant in the *CD36* gene. She has a female sibling with the same condition, who has not been genetically tested

### Thrombocytopenia

Four patients with persistent thrombocytopenia were evaluated. Two patients (50%) had VUSs in relevant genes. One patient with an ADAMTS13 gene mutation did not exhibit low ADAMTS13 levels and did not respond to any treatment for thrombocytopenia, except for splenectomy Table 4.

**Table 4:** Genetic testing results and clinical findings in patients with thrombocytopenia

	Gene	Gender	Clinical presentation	variant	OMIM phenotype	ACMG classification	zygosity	Mode of inheritance	Lab
P29	ADAMTS13	F	Persistent thrombocytopenia and bleeding tendency	c.686 + 60G > A c.1308 + 109G > A	Thrombotic thrombocytopenic purpura hereditary	VUS	Het	AR	AiLife
P30	CFI	F	thrombocytopenia	c.772 + 20 C > G	Complement factor I deficiency, Hemolytic uremic syndrome atypical 3	VUS	Het	AD	AiLife
P31	TOM1	F	Thrombocytopenia	c.899G > A (p.Arg300Gln)	Immunodeficiency 85 and autoimmunity	VUS	Het	AD	AiLife

### Stroke

Five patients with stroke underwent WES. A definitive diagnosis was provided to two patients (40%). The remaining three patients (60%) had VUSs in genes relevant to the clinical phenotype, and further conventional testing is needed to confirm the pathogenicity of the variants Table 5.

**Table 5:** Genetic testing results and clinical findings in patients with stroke

	Gene	Gender	Clinical presentation	variant	ACMG classification	OMIM phenotype	Zygosity	Mode of inheritance	Lab
P32	GLA	M	Stroke in a 10-year-old boy	c.1055 C > G (*p*.Ala352Gly)	VUS	Fabry disease	Hemizygous	XL	AiLife
P33	HBB	F	Stroke and seizures	c.20 A > T(p.Glu7Val)	P	Sickle cell disease (SCD)	Hom	AR	AiLife
P34	COL4A1	M	Stroke	c.1382-86G > A	VUS	Hereditary angiopathy, Brain small vessel disease	Het	AD	AiLife
P35	SCN2A HBB	F	SCD with recurrent ataxia	c.2177 C > T (p.Pro726Leu) c.20 A > T (p.Glu7Val)	VUS P	Episodic ataxia type 9 Sickle cell disease	Het Hom	AD AR	AiLife
^1^P36	F11	F	Stroke	c.1078 C > G (p.Leu360Val)	VUS	Factor XI deficiency, autosomal dominant	Het	AD	AiLife

^1^ Although F11 deficiency is typically linked to a lower risk of stroke, this patient still experienced one

### Cytosis

Seven patients suspected of having a bone marrow proliferative disorder underwent genetic testing via WES. Six patients (85%) had LP variants, leading to a definitive molecular diagnosis through WES alone. P43 had a VUS in a gene associated with the phenotype Table 6.

**Table 6:** Genetic testing results and clinical findings in patients with cytosis

	Gene	Gender	Clinical presentation	Variant	ACMG classification	OMIM phenotype	Zygosity	Mode of inheritance	Lab
^1^P37	MPL	M	Thrombocytosis	c.317 C > T (*p*.Pro106Leu)	LP	Thrombocythemia 2	Hom	AD	AiLife
^1^P38	MPL	M	Thrombocytosis	c.317 C > T (p.Pro106Leu)	LP	Thrombocythemia 2	Hom	AD	AiLife
P39	SLC30A10	M	Polycythemia, dystonia	c.1006 C > T (p.His336Tyr)	LP	Hypermanganesemia with dystonia 1	Hom	AR	Revvity
^2^P40	MEFV	F	Abdominal pain edema allergy and severe hyperesino philia	c.2177T > C (p.Val726Ala)	LP	Familial Mediterranean fever (FMF), Neutrophilic dermatosis	Het	AD	AiLife
^2^P41	MEFV	F	Abdominal pain edema allergy and severe hyperesino philia	c.2177T > C (p.Val726Ala)	LP	FMF, Neutrophilic dermatosis	Het	AD	AiLife
^3^P42	STAT1	F	Severe hypereosinophilia and a cytomegalovirus (CMV) infection	c.1760_1761del (p.Glu587AlafsTer18)	LP	Immunodeficiency 31B mycobacterial and viral infections	Hom	AR	AiLife
P43	CYB5R3	M	Cyanosis, poor school performance, polycythemia	c.640_642del (p.Lys214del)	VUS	Methemoglobinemia type II	Hom	AR	AiLife

^1^ Two unrelated Saudi patients with thrombocytosis were found to have the same variant in the MPL gene

^2^ Two Egyptian sisters with severe hypereosinophilia and skin rashes exhibited the same variant in the MEFV gene

^3^ A Saudi female infant presenting with severe hypereosinophilia, bronchopneumonia, and a high CMV viral load was found to have a STAT1 gene variant

### Hemolytic anemia (HA)

Among the twelve patients who presented with HA, nine patients (75%) received a molecular diagnosis through WES alone. Three patients (25%) had VUSs in clinically relevant genes. Notably, six patients (50%) were diagnosed with pyruvate kinase deficiency (PKD) (Table 7).

**Table 7:** Genetic testing results and clinical findings in patients with hemolytic anemia

	Gene	Gender	Clinical presentation	Variant	ACMG classification	OMIM phenotype	Zygosity	Mode of inheritance	Lab
P44	SPTB	M	HA and splenomegaly	c.2352_2353delinsT (*p*.Lys784AsnfsTer15)	LP	Hereditary spherocytosis	Het	AD	AiLife
P45	NT5C3A	M	HA	c.497G > T (p. Gly166Val)	VUS	Hemolytic anemia due to UMPH1 deficiency	Hom	AR	Revvity
P46	PKLR SLCO1B1 SLCO1B3	M	HA and direct hyperbilirubinemia	c.858del (p.Phe287LeufsTer34) c.1738 C > T (p.Arg580Ter) c.628 + 87T > G	LP P VUS	PKD Hyperbilirubinemia Rotor type Hyperbilirubinemia Rotor type	Hom Hom Hom	AR AR AR	AiLife
P47	HBB	M	HA and HSM	c.289_290delinsGG (p.Leu97Gly)	VUS	Unstable hemoglobinopathies	Het	AD	Revvity
P48	RFXANK	M	Autoimmune HA with lymphopenia	c.362 A > T (p.Asp121Val)	P	MHC class II deficiency, complementation group B	Hom	AR	Revvity
P49	PKLR	F	HA	c.1436G > A (p.Arg479His)	P	PKD	Hom	AR	AiLife
P50	PKLR	M	HA	c.1436G > A (p.Arg479His)	P	PKD	Hom	AR	Revvity
P51	PKLR	M	HA	c.1528 C > T (p.Arg510Ter)	P	PKD	Hom	AR	Revvity
P52	PKLR	F	HA	c.1436G > A (p.Arg479His)	P	PKD	Hom	AR	Revvity
P53	PKLR ALG3	F	HA and Dandy Walker syndrome	c.1436G > A (p.Arg479His) c.626T > C (p.Met209Thr),	P VUS	PKD Congenital disorder of glycosylation type Id	Hom Hom	AR AR	AiLife
P54	ANK1	Male	HA	c.1436G > A (p.Arg479His)	VUS	Spherocytosis, type 1	Het	AD	Revvity

### Non-hemolytic anemia

Four patients with non-hemolytic anemia were tested. One patient (25%) received a definitive molecular diagnosis. The remaining three patients (75%) had VUSs in clinically relevant genes (Table 8).

**Table 8:** Genetic testing results and clinical findings in patients with non- hemolytic anemia

	Gene	Gender	Clinical presentation	Variant	ACMG classification	OMIM phenotype	Zygosity	Mode of inheritance	Lab
P55	RPL15 ALDH7A1	M	Severe non-hemolytic anemia and severe intractable seizures.	c.587 A > G (*p*.Asn196Ser) c.1514del (*p*.Gly505AlafsTer13)	VUS LP	Diamond-Blackfan anemia 12 Epilepsy pyridoxine-dependent	Het Hom	AD AR	AiLife
P56	HEATR3	M	Persistent macrocytic anemia, thrombocytosis	c.934 A > G (p.Ile312Val)	VUS	Diamond-Blackfan anemia 21	Het	AD	AiLife
P57	22q12.3 copy loss “TST, MPST, KCTD17 and TMPRSS6”	F	Iron deficiency anemia not responsive to therapy	Copy variant loss	LP	Autosomal recessive iron-refractory iron deficiency anemia	Hom	AR	Revvity
P58	STIM1 ADA2	F	Severe anemia	c.1439T > C (p.Met480Thr) “c.342T > G (p.His114Gln)	VUS VUS	Immunodeficiency 10 Sneddon syndrome	Het Hom	AD AR	Revvity

Notaboly, 12 patients (17%) received a molecular diagnosis of an inborn error of immunity. Among these immunological diagnoses, six patients (P1, P6, P22, P30, P31, P48, P58) with cytopenia were identified as having immunodeficiency (ID)-related variants, whereas two patients with neutropenia (P2, P8) were found to have variants linked to autoinflammation. Furthermore, among the patients with severe hypereosinophilia, two were diagnosed with FMF and one was found to have an ID.

An additional eight patients (11%) were diagnosed with non-hematological conditions, including two patients with Klinefelter syndrome and one patient each with Fabry disease, Barth syndrome, MPS type 6, vasculopathy, episodic ataxia, and hypermagnesemia with dystonia.

**Table 9:** Diagnostic yield of WES by hematological disorder subtype

Hematological disorder subgroup	Number of cases (*n*)	Number of cases with positive WES diagnosis	Success rate (%)
Neutropenia	11	2	18
Cytopenia	14	7	50
Bleeding tendency	7	3	42
Thrombocytopenia	3	0	0
Stroke	5	2	40
Cytosis	7	6	85
Hemolytic anemia	12	9	75
Non-hemolytic anemia	4	1	25

## Discussion

This study highlights the experiences of a single center in SA that employs WES to diagnose ambiguous hematological presentations in pediatric patients. A total of 67 patients presenting with symptoms of hematological disorders underwent testing. The diagnostic yield of WES in this study was 48%. As shown in Table 9, the yield varied across hematological disorder subtypes. In comparison, a previous study by Retterer et al. reported a lower diagnostic rate of 26%, which may be attributed to the smaller sample size (*n* = 19) and the specific criteria used for patient selection [3]. Similarly, Yang Y et al. studied a large cohort of approximately 2000 patients referred for evaluation of suspected genetic conditions and reported a diagnostic yield of 25%, highlighting variability in WES diagnostic rates depending on cohort characteristics [4]. 

In our study, 38% of the patients had VUSs in genes relevant to their clinical phenotype. This aligns with the findings of Elain Chen et al., who reported VUSs in 41% of the 1.6 million individuals undergoing molecular testing, with the highest rates observed among individuals of non-European White descent [5]. Notably, most patients in our cohort were Arab and Middle Eastern in ethnicity.

Moreover, the Genome-Wide Association Study (GWAS) Catalog indicates that 88% of individuals are of European ancestry, whereas only 0.17% represent Arab populations, making them among the least represented groups [6]. This limited representation complicates the interpretation of genetic variants and may contribute to the high percentage of VUSs observed in our cohort.

It is worth noting that these VUSs would have required additional testing—such as Sanger sequencing, functional assays, or familial segregation studies—to confirm their pathogenicity. However, none of these confirmatory tests were performed as part of this study. The absence of such follow-up investigations may have contributed to an underestimation of the true diagnostic yield.

In our study, the diagnostic rate for neutropenic patients was 18%, which is lower than the 62.5% reported by Grigorios Tsaknakis et al. in a study of 16 adults. Their higher diagnostic rate may be attributed to the use of a specialized in-house bioinformatics pipeline for analysing WES data, which specifically targets genes associated with phagocyte defects, bone marrow failure, and immune disorders [7]. In contrast, we outsourced our WES samples to clinical laboratories, where analysis methods are not standardized, making it more difficult to interpret the results. In addition, 63% of our neutropenic patients had VUSs.

This study identified specific subtypes of neutropenia, including syndromic, monogenic, autoinflammatory, and ID-associated forms. Achieving a precise diagnosis in neutropenic patients enables personalized treatment, such as the use of granulocyte colony-stimulating factor (G-CSF) or hematopoietic stem cell transplantation (HSCT). Additionally, it enhances risk monitoring for complications such as malignancies and provides valuable information for genetic counseling.

In this study, applying WES after a comprehensive conventional diagnostic workup to cases with suspected IBMFS achieved a diagnostic rate of 46%, surpassing the 35% rate reported by Motoharu Hamada et al., who used a capture-based targeted sequencing approach covering over 180 related genes [8]. 

Diagnosing IBMFS can be challenging owing to their overlapping and atypical presentations, driven by variable penetrance. For example, a subset of dyskeratosis congenita (DC) with AD inheritance may present later in adolescence or adulthood. This explains the non-classic phenotype observed in three children with DC in this study.

Accurate diagnosis of IBMFS is crucial, as these syndromes are associated with an elevated risk of solid tumors and hematopoietic malignancies, necessitating robust cancer surveillance. Furthermore, distinguishing between inherited and acquired bone marrow failure syndromes is essential for guiding treatment decisions.

Identifying the IBMFS before HSCT is particularly important. It enables tailored adjustments to the HSCT conditioning regimen and helps avoid the use of family donors who may carry the same genetic mutation, ultimately improving patient outcomes [9, 10]. 

In this study, three monogenic disorders—Fabry disease, COL4A1-related syndromes, and SCD—were identified in 80% (4 out of 5) of patients who presented with stroke. Similarly, a retrospective analysis of 124 patients who underwent WES for major stroke reported that 8.9% received a definitive diagnosis, whereas 10.5% had a probable diagnosis [11]. 

Early recognition of stroke and its risk factors in children is crucial, as it enables timely implementation of neuroprotective, thrombolytic, and antithrombotic treatments, along with post-stroke rehabilitation strategies. For instance, one patient with SCD who experienced recurrent ataxia was found to have an SCN2A gene mutation through WES, leading to significant changes in the management plan.

Although some authors advocate for the use of stroke gene panels, the understanding of monogenic disorders associated with stroke risk continues to evolve, resulting in dynamic updates to the genes included in these panels. For example, Andreea Ilinca et al. expanded their stroke gene panel from 168 to 240 genes over 4.5 years [12]. This progress supports the use of WES as a first-tier molecular testing method for patients presenting with stroke, rather than relying exclusively on fixed gene panels.

The complex homeostatic process is regulated by various genetic factors. Physicians often rely on traditional coagulation tests, which have inherent limitations and can be affected by numerous variables, often leading to delayed or inconclusive diagnoses.

In our study, incorporating WES provided a definitive diagnosis in 42% of patients with bleeding tendencies. Additionally, 42% of patients had VUSs in genes associated with their rare disorders. However, the lack of further confirmatory testing limited the diagnostic yield, which could have potentially increased to 84%.

Similarly, a cross-sectional study conducted in the Netherlands demonstrated the utility of WES, with a diagnostic yield of 85% (132 out of 156 patients). In that study, 24% of the 214 identified rare genetic variants were classified as VUSs [13]. 

Our study highlights the value of WES in diagnosing HA, with 75% (9 out of 12) of patients receiving a molecular diagnosis. In this context, Hendilli et al. demonstrated the utility of WES in identifying genetic causes of HA, achieving a diagnostic rate of 82.5% (33 out of 40 patients) [14]. The latest Korean clinical practice guidelines for diagnosing hereditary hemolytic anemia (HHA) recommend using NGS as the first diagnostic step, before analysing red blood cell membrane proteins and enzymes [15]. 

Identifying genetic mutations is critical for selecting appropriate management strategies for HA. For example, studies have shown that patients with specific genetic variants in PKD [16] and hereditary spherocytosis [17] may respond poorly to splenectomy. In such cases, alternative treatments such as chronic transfusions, chelation therapy, or HSCT should be considered.

Our findings show that PKD is a frequent cause of undiagnosed hemolysis, representing 6 out of 12 cases in our study. Similarly, Russo et al., using a targeted NGS panel, reported that 45.5% of probands initially diagnosed with congenital dyserythropoietic anemia (CDA) were reclassified as having PKD due to mutations in the PKLR gene [18]. 

In this study, 12 patients (17%) were diagnosed with inborn errors of immunity, a diverse group of disorders that can present early with autoimmune cytopenia. These conditions include ID, autoinflammation, lymphoproliferation, and malignancy. Advances in genetic testing for these disorders have facilitated the repurposing of targeted immunological therapies, significantly expanding the available treatment options [19]. 

Confirming a patient’s phenotype through traditional laboratory tests remains vital, as genetic causes may still go undetected despite advances in sequencing technologies [19]. For example, WES failed to identify variants in 8 cases (11%), and VUSs were detected in 26 cases (38%). Furthermore, discrepancies between genotype and phenotype were noted in patients P2, P21, and P29. These findings emphasize the limitations of WES in certain cases and the challenges of interpreting genetic data in the context of clinical phenotypes. While some VUS findings may have contributed to provisional diagnoses or informed clinical decision-making, their interpretation remains inherently limited without additional supporting evidence. The absence of confirmatory functional assays or familial segregation studies further restricts our ability to establish definitive pathogenicity. Therefore, we emphasize the importance of incorporating complementary genetic, functional, and segregation analyses to validate the clinical relevance of VUS and to prevent overinterpretation in both research and diagnostic settings.

Clinicians have several genetic testing options, including targeted NGS panels, WES, and whole-genome sequencing (WGS). Targeted panels are most effective when there is a well-defined phenotype and the panel comprehensively covers the relevant genes. However, for many hematological and immunodeficiency disorders, where phenotypes can be complex and heterogeneous, broader approaches such as WES or WGS may be needed to identify the underlying genetic causes. Therefore, when selecting the most suitable genetic testing method, clinicians should carefully evaluate patients’ clinical and laboratory phenotypes, as well as their family history [20, 21]. 

## Conclusion

In conclusion, this study highlights the growing role of genomic testing in the evaluation of pediatric hematological disorders. However, the effective interpretation and clinical application of genetic findings require close collaboration between pediatricians and geneticists. An integrated, multidisciplinary approach is essential to ensure accurate diagnosis, optimize patient management, and facilitate informed genetic counseling. Strengthening this collaboration will not only enhance diagnostic yield but also improve the overall quality of care for affected children and their families.

### Study Limitations

This study has several limitations that should be considered when interpreting the findings. First, the sample size was relatively small. Second, the study relied on retrospective data collection, which may limit the accuracy of the information gathered. Third, a major limitation was the reliance on singleton WES, as parental samples were not routinely available due to logistical, financial, and clinical factors at the time. This limited our ability to perform segregation analysis and confidently interpret certain variants, particularly VUSs. As a result, comprehensive genetic counseling and recurrence risk assessment were not always possible. Future studies should prioritize trio or dual WES to enhance diagnostic accuracy and improve clinical utility.Fourth, the ethnic background of the participants was predominantly Saudi, which restricts the generalizability of the findings to other ethnic groups. Fifth, data on parental consanguinity were not consistently available in the medical records and could not be included in the analysis. This limits our ability to assess its potential contribution to the observed genetic findings. Sixth, Detailed data on the clinical consequences of WES findings were not available, as this was a retrospective study and many patients were referred to higher-level centers for ongoing care. As a result, our analysis focused solely on diagnostic yield rather than post-diagnostic management or outcomes. Finally, confirmatory analyses—including Sanger sequencing, functional assays, and familial studies—were not performed, which restricted our ability to validate potentially pathogenic variants and establish definitive genotype–phenotype correlations. The absence of these follow-up investigations may have led to an underestimation of the diagnostic yield. Future studies should aim to incorporate trio or duo WES and follow-up confirmatory testing to improve diagnostic accuracy and clinical utility.

## Data Availability

The datasets used in the current study are available from the corresponding author upon reasonable request.

## Abbreviations

WES: Whole exome sequence
EMRs: Electronic medical records
MCH: Maternity and Children Hospital
SA: Saudi Arabia
IRB: Institutional review board
NGS: Next generation sequence
CNVs: Copy number variants
SNVs: Single nucleotide variants
BWA: Burrows-Wheeler aligner
GATK: Genome analysis toolkit
ACMG: American College of Medical Genetics guidelines
P: Pathogenic
LP: Likely pathogenic
VUS: Variant of unknown significance
XLR: X-linked recessive
M: Male
F: Female
AR: Autosomal recessive
AD: Autosomal dominant
XL: X-linked
Hom: Homozygous
Het: Heterozygous
HSM: Hepatosplenomegaly
MPS: Mucopolysaccharidosis
PTT: Partial thromboplastin time
SCD: Sickle cell disease
FMF: Familial Mediterranean fever
CMV: Cytomegalovirus
HA: Hemolytic anemia
ID: Immunodeficiency
HSCT: Hematopoietic stem cell transplantation
IBMFS: Inherited bone marrow failure syndromes
DC: Dyskeratosis congenita
WGS: Whole-genome sequencing
